# Patient characteristics but not virulence factors discriminate between asymptomatic and symptomatic *E. coli* bacteriuria in the hospital

**DOI:** 10.1186/1471-2334-13-213

**Published:** 2013-05-10

**Authors:** Jonas Marschall, Marilyn L Piccirillo, Betsy Foxman, Lixin Zhang, David K Warren, Jeffrey P Henderson

**Affiliations:** 1 Division of Infectious Diseases, Washington University School of Medicine, 660 S. Euclid, St. Louis 63110, MO, USA; 2 School of Public Health, University of Michigan, Ann Arbor, MI, USA; 3 Department of Infectious Diseases, Bern University Hospital and University of Bern, Friedbühlstr. 51, 3010 Bern, Switzerland

**Keywords:** Escherichia coli, Bacteriuria, Urinary tract infection, Asymptomatic, Virulence factors

## Abstract

**Background:**

*Escherichia coli* is a common cause of asymptomatic and symptomatic bacteriuria in hospitalized patients. Asymptomatic bacteriuria (ASB) is frequently treated with antibiotics without a clear indication. Our goal was to determine patient and pathogen factors suggestive of ASB.

**Methods:**

We conducted a 12-month prospective cohort study of adult inpatients with *E. coli* bacteriuria seen at a tertiary care hospital in St. Louis, Missouri, USA. Urine cultures were taken at the discretion of treating physicians. Bacterial isolates were tested for 14 putative virulence genes using high-throughput dot-blot hybridization.

**Results:**

The median age of the 287 study patients was 65 (19–101) years; 78% were female. Seventy percent had community-acquired bacteriuria. One-hundred ten (38.3%) patients had ASB and 177 (61.7%) had symptomatic urinary tract infection (sUTI). Asymptomatic patients were more likely than symptomatic patients to have congestive heart failure (p = 0.03), a history of myocardial infarction (p = 0.01), chronic pulmonary disease (p = 0.045), peripheral vascular disease (p = 0.04), and dementia (p = 0.03). Patients with sUTI were more likely to be neutropenic at the time of bacteriuria (p = 0.046). Chronic pulmonary disease [OR 2.1 (95% CI 1.04, 4.1)] and dementia [OR 2.4 (95% CI 1.02, 5.8)] were independent predictors for asymptomatic bacteriuria. Absence of pyuria was not predictive of ASB. None of the individual virulence genes tested were associated with ASB nor was the total number of genes.

**Conclusions:**

Asymptomatic *E. coli* bacteriuria in hospitalized patients was frequent and more common in patients with dementia and chronic pulmonary disease. Bacterial virulence factors could not discriminate symptomatic from asymptomatic bacteriurias. Asymptomatic *E. coli* bacteriuria cannot be predicted by virulence screening.

## Background

*Escherichia coli* is the most frequent pathogen to cause symptomatic urinary tract infection (sUTI) but can also lead to asymptomatic bacteriuria. Asymptomatic bacteriuria (ASB) denotes bacterial colonization of the urogenital tract without subjective or systemic host responses. ASB screening and treatment are recommended only during pregnancy [1] and in the preoperative evaluation of men before urological procedures [2], circumstances where preemptive antibiotic administration decreases the risk of infectious complications [3]. Despite these recommendations, overuse of antibiotics for ASB is common and clearance of bacteriuria is often transient, leading to further treatment courses [4]. Inappropriate antibiotics result in increased healthcare costs and foster antimicrobial resistance [5]; they can even eliminate the protective effect that ASB may have against recurrent UTIs [6]. These issues have led to calls for assessing hospitals’ performance in reducing inappropriate antibiotic use nationwide [7].

Surprisingly few studies have compared patients with symptomatic bacteriuria to patients with ASB to identify patient-level risk factors for symptomatic presentation or differences in bacterial virulence factors [8-12]. Results from these studies have been inconsistent, reporting either a variety of virulence factors associated with symptomatic bacteriuria [8-11] or no association [12]. We found no previous studies addressing clinical risk factors. Urine cytokines such as interleukin 6 and 8 may be lower in ASB in certain populations [13,14], but an objective test to discriminate ASB from symptomatic bacteriuria is not commercially available. A better understanding of what differentiates ASB from symptomatic bacteriuria could be particularly helpful in patients who are unable to report symptoms, such as intubated patients or those with altered mental status. Here, we used a prospective cohort of patients with *E. coli* bacteriuria as identified by clinicians in a hospital setting. The purpose of our study was to characterize host and pathogen factors associated with *E. coli* ASB that could subsequently be used in predictive models, lead to the development of bedside tests to distinguish ASB from sUTI, or guide treatment decision-making.

## Methods

### Study design, data collection, and definitions

We performed a prospective cohort study of patients with *E. coli* bacteriuria from August 1st 2009 until July 31st, 2010, at Barnes-Jewish Hospital (BJH), a 1250-bed teaching hospital in eastern Missouri. All adult patients admitted to BJH who presented with *E. coli* bacteriuria at time of admission or developed it subsequently were considered for enrollment. Urine cultures were taken at the discretion of the treating physician. The cut-off for significant bacteriuria employed in our hospital microbiology laboratory was 5×10^4^ colony-forming units/ml in non-catheterized and 5×10^3^ in catheterized patients. The bacteriuria was classified as community-acquired if the first positive urine culture occurred within 48 hours of admission. Patients transferred from outside hospitals or long-term care facilities were not considered to have community-acquired bacteriuria. Polymicrobial UTIs were excluded, as were patients who had concurrent bloodstream infection with an organism other than *E. coli*. We reviewed medical records of those who met inclusion criteria for demographics, medical and urogenital history, and computed Charlson comorbidity and McCabe severity of illness scores. The patients’ clinical, laboratory, radiological, and pharmacy data were prospectively reviewed during the admission, including information on all urine and blood cultures. Medication information was entered as start and stop dates and times for each antibiotic with Gram-negative activity.

We classified *E. coli* bacteriurias based on the patients’ urinary symptoms with the objective of determining distinctive features of asymptomatic bacteriuria (ASB). ASB was defined as absence of urinary symptoms. Symptomatic urinary tract infection (sUTI) included cystitis, defined as presence of dysuria, frequency, urinary retention, or lower abdominal pain (without signs of pyelonephritis); or pyelonephritis, defined as the presence of flank pain or tenderness and/or fever. We defined *unclassifiable bacteriuria* as bacteriuria in a patient who did not fit any of the above criteria or could not report symptoms (e.g., intubation, altered mental status); these patients were excluded from the analysis. If a urinary catheter had been in place in the 48 hours preceding the positive urine culture the bacteriuria was considered as catheter-associated. Past urogenital surgery included all surgeries that resulted in anatomical alteration (e.g. nephrectomy, neobladder formation, prostatic resection, and hysterectomy).

Outcomes of interest were sepsis, sepsis-induced hypotension, *E. coli* bacteremia, transfer to the ICU within 72 hours of the bacteriuria, length of hospital stay after detection of bacteriuria, and in-hospital mortality. Sepsis and sepsis-induced hypotension were defined using established criteria [15]. Blood cultures were drawn at the discretion of the treating physician and had to occur within ±1 day of the bacteriuria. Adequacy of antibiotic therapy was defined as pathogen-directed treatment with antibiotics matching susceptibilities.

### Laboratory analyses

We identified the *E. coli* isolates from urine cultures of eligible patients in the hospital microbiology laboratory and stored them at -80°C in skim milk. Isolates were processed at the Center for Molecular and Clinical Epidemiology at the School of Public Health, University of Michigan, Ann Arbor, MI. Bacterial DNA was extracted using QIAamp DNA mini kit (QIAGEN, Valencia, CA). DNA probes for the following virulence genes were designed: Hemolysin (*hlyA*), P family of fimbriae (*prf*) [this primer detects all *pap* adhesions], Dr family of adhesins (*Dr*), S fimbriae (*sfaS*), cytotoxic necrotizing factor (*cnf1*), aerobactin iron uptake system (*iutA*), salmochelin iron uptake system (*iroN*), yersiniabactin iron uptake system (*fyuA*), group II capsule gene (*kpsMT*), irgA homologue adhesin (*iha*), uropathogenic specific protein (*usp*), outer membrane protein (*ompT*), the secreted autotransporter toxin (*sat*), and for *chuA* (outer heme receptor). The presence of these virulence genes was determined by dot-blot hybridization with fluorescent-labeled probes and a fluorescein-based detection system as described elsewhere [16]. To screen large numbers of isolates in short time periods, we used a microarray system for high-throughput dot-blot hybridization [17]. The frequency of specific virulence genes was compared between symptomatic and asymptomatic patients. Furthermore, bacterial isolates were compared regarding antimicrobial susceptibility patterns.

### Sample size calculations and statistical analysis

We based our sample size calculations on two studies that reported 34-44% of bacteriuric patients had symptoms and that males were slightly more likely to have sUTI [8,18]. Assuming that male patients account for 65% in the sUTI and for 45% in the ASB group and given a 0.05 significance level and 80% power, we estimated that we would require 88 vs. 132 patients in the two groups, respectively (EpiInfo 3.3.2; accessed at http://www.cdc.gov/epiinfo/).

Data analysis was performed using SPSS 18 (SPSS Inc., Chicago, IL). Univariate comparisons among categorical variables were performed using the χ^2^ test or Fisher’s exact test as appropriate. Comparisons among continuous independent variables were performed using Student’s t test or Mann Whitney U test as appropriate. A two-sided *p* value of <0.05 was considered significant. Variables found to have a *p* ≤ 0.1 on univariate testing were simultaneously entered into a multivariate logistic regression model. The model was tested with goodness-of-fit measures.

The study was approved both by the Washington University Human Research Protection Office and the University of Michigan Institutional Review Board. We obtained a waiver of informed consent from both Boards.

## Results

We identified 337 patients with *E. coli* bacteriuria during the study period. Of these, 50 (14.8%) were excluded because they were considered unclassifiable (e.g., intubated patients or patients with acutely altered mental status who were unable to report symptoms). Among the remaining 287 patients, 110 (38.3%) were classified as having asymptomatic bacteriuria, and 177 (61.7%) as having symptomatic UTI. Two hundred and twenty-five patients were female (78.4%), and 169 (58.9%) were white (Table 1). Age had a bimodal distribution, with a small peak between 20 and 25 years and a larger peak between 60 to 80 years; the median age was 65 years (range, 19–101). Among symptomatic patients, 70 (39.4%) had cystitis, and 107 (61.6%) pyelonephritis. In this group of symptomatic patients, 91 (51.4%) were tested for bloodstream infection, and 20 had positive blood cultures. Among asymptomatic patients, 35 (31.8%) had blood cultures taken, and one was positive for *E. coli*.

**Table 1:** **Comparison of 110 patients with asymptomatic bacteriuria to 177 patients with symptomatic urinary tract infection due to *E. coli***

**Variable**	**Asymptomatic bacteriuria (n = 110)**	**Symptomatic urinary tract infection (n = 177)**	**p value**	Adjusted odds ratio (95% CI)*
	n (%)	n (%)		
Gender (male)	19 (17)	43 (24)	0.2	
Age (years, median, range)	67 (20, 100)	64 (19, 101)	0.1	
Race (white)	68 (62)	101 (57)	0.4	
Body mass index (kg/m^2^, mean, ±SD)	28.9 (±8.4)	28.9 (±9.9)	1.0	
Diabetes mellitus	39 (36)	53 (30)	0.3	
Renal insufficiency (Cr > 1.5 mg/dl)	23 (21)	37 (21)	1.0	
Any malignancy	28 (26)	44 (25)	0.9	
Any transplant	3 (3)	9 (5)	0.3	
Neutropenia at time of bacteriuria (ANC <1000/ul)	0	7 (4)	0.046	
Pregnancy	5 (5)	8 (6)	1.0	
Congestive heart failure	26 (24)	24 (14)	0.03	
Myocardial infarction	23 (21)	18 (10)	0.01	
Chronic pulmonary disease	25 (23)	24 (14)	0.045	2.1 (1.04-4.1)
Peripheral vascular disease	9 (8)	5 (3)	0.04	
Oral steroid medication	6 (5)	15 (8)	0.3	
Dementia	15 (14)	11 (6)	0.03	2.4 (1.02-5.8)
History of cerebrovascular accident	24 (22)	36 (20)	0.8	
Hemi- or paraplegia	13 (12)	14 (8)	0.3	
Functional or anatomical urinary tract abnormalities	31 (28)	54 (31)	0.7	
Voiding dysfunction	26 (24%)	54 (31%)	0.2	
Benign prostatic hyperplasia (n = 62)	3/19 (16)	15/43 (35)	0.1	
History of urogenital surgery	30 (27)	61 (34)	0.8	
Urological procedure this admission	0	5 (3)	0.2	
Charlson comorbidity index (mean, ±SD)	3.1 (±2.6)	2.8 (±2.8)	0.2	
McCabe severity-of-illness score (median, range)	1 (1, 2)	1 (1, 3)	0.2	
Cystitis	-	70 (39)	-	
Pyelonephritis	-	107 (62)	-	
Sepsis	38 (35)	108 (61)	<0.001	
Sepsis-induced hypotension	7 (6)	41 (23)	<0.001	
Community-acquired bacteriuria	75 (68)	127 (72)	0.5	
Urinary catheter-associated bacteriuria	22 (20)	30 (17)	0.5	
Urinalysis with pyuria (>10 WBC/hpf)	65 (63)	121 (74)	0.1	
Isolate resistant to ciprofloxacin	57 (32%)	41 (37%)	0.4	
Isolate resistant to trimethoprim/ sulfamethoxazole	45 (25%)	32 (29%)	0.5	
***Outcomes***				
Pathogen-directed antibiotic treatment	89 (81)	168 (95)	<0.001	
Length of hospital stay (mean, ±SD)	5.5 (±7.7)	6.6 (±7.5)	0.2	
In-hospital mortality	4 (3.6)	8 (5)	0.5	

NOTE. *CI *= confidence interval. *SD *= standard deviation. *Cr *= creatinine. *ANC *= absolute neutrophil count. *WBC *= white blood cells. *HPF *= high-power field.

* The variables included in the final model were neutropenia, congestive heart failure, chronic pulmonary disease, history of myocardial infarction, peripheral vascular disease, dementia, and pyuria.

### Comparison of patient risk factors for asymptomatic bacteriuria

Asymptomatic patients were more likely than symptomatic patients to have congestive heart failure (p = 0.03), a history of myocardial infarction (p = 0.01), chronic pulmonary disease (p = 0.045), peripheral vascular disease (p = 0.04), and dementia (p = 0.03) (Table 1). Conversely, patients with symptomatic UTI were more likely to be neutropenic at the time of bacteriuria (p = 0.046). Subsets of catheter-associated bacteriuria were similar in the two groups (p = 0.5). Also, there was no difference in the subset of *complicated* bacteriurias between the groups [95 (86%) among asymptomatic vs. 153 (86%); p = 1.0].

### Comparison of virulence factors for asymptomatic bacteriuria

We also compared the prevalence of virulence genes in the bacterial isolates from both groups (see Table 2). The most frequently detected virulence factors were *chuA* (83.3%), *fyuA* (82.9%), and *ompT* (79.8%). There were no statistically significant differences encountered. The total number of virulence factors per isolate did not differ between asymptomatic [median 6.5 (range, 0–13)] and symptomatic [median 7 (0–12)] patients (p = 0.2).

**Table 2:** **Comparison of virulence genes present in *E. coli *isolates from 287 patients with asymptomatic bacteriuria or symptomatic urinary tract infection**

**Variable**	**Asymptomatic bacteriuria (n = 110)**	**Symptomatic urinary tract infection (n = 177)**	**p value**
	n (%)	n (%)	
*chuA*	90 (81.8)	149 (84.2)	0.6
*fyuA*	91 (82.7)	147 (83.1)	0.9
*ompT*	83 (75.5)	146 (82.5)	0.15
*usp*	79 (71.8)	117 (66.1)	0.3
*kpsMT* (group II capsule)	59 (53.6)	95 (53.7)	1.0
*iucD*	49 (44.5)	89 (50.3)	0.3
*iha*	51 (46.4)	70 (39.5)	0.3
*sat*	43 (39.1)	75 (42.4)	0.6
*prf* (P family of fimbriae)	28 (25.5)	51 (28.8)	0.5
*iroN*	25 (22.7)	40 (22.6)	1.0
*hlyA*	19 (17.3)	38 (21.5)	0.4
*sfa*	20 (18.2)	30 (16.9)	0.8
*cnf1*	13 (11.8)	20 (11.3)	0.9
*Dr*	6 (5.5)	14 (7.9)	0.4

When comparing the resistance pattern of bacterial isolates there was no difference in resistance to the most frequently used antibiotics ciprofloxacin [41/110 (37%) among sUTI vs. 57/177 (32%) among ASB patients; p = 0.4] or trimethoprim/sulfamethoxazole [32/110 (29%) vs. 45/177 (25%); p = 0.5].

### Multivariate analysis of risk factors for asymptomatic bacteriuria

We performed a multivariate analysis of risk factors for ASB entering variables that had a *p* ≤0.1 in univariate analysis. Based on the numbers of ASB cases we intended to include a maximum of 10–12 variables into the model. Neutropenia, congestive heart failure, chronic pulmonary disease, history of myocardial infarction, peripheral vascular disease, dementia, and pyuria were entered into the model. Chronic pulmonary disease [OR 2.1 (95% CI 1.04, 4.1)] and dementia [OR 2.4 (95% CI 1.02, 5.8)] were independent predictors for asymptomatic bacteriuria. Pyuria was not associated with symptomatic UTI. The Hosmer-Lemeshow test indicated a good fit for the data (p = 0.3).

### Clinical outcomes

There was no difference in the proportion of patients requiring admission to the intensive care unit between the two groups [11 (10%) ASB vs. 22 (12%) sUTI; p = 0.5]. Symptomatic UTI patients were more likely to meet sepsis criteria [108 (61%) vs. 38 (35%); p < 0.001] and develop sepsis-induced hypotension [41 (23%) vs. 7 (6%); p < 0.001]. Twelve patients died during their hospital admission (4.2%). There was no difference in mortality (p = 0.5) nor in length of hospital stay after the bacteriuria whether it was symptomatic or not (p = 0.2).

Fewer ASB patients received pathogen-directed antibiotic treatment during the hospitalization [89 (81%) vs. 168 (95%); p < 0.001]. Among those receiving antibiotic treatment, the mean time from detection of bacteriuria to receipt of appropriate antibiotics was shorter for symptomatic patients [15.9 hours (±26.8) vs. 22.4 hours (±23.2); p = 0.07). We did, however, not compare treatment variation in the antibiotic therapy as part of this analysis and antibiotic prescriptions at hospital discharge were not taken into account.

## Discussion

Why *E. coli* bacteriuria is associated with urinary tract symptoms in some instances but not in others remains incompletely understood. This knowledge gap may explain why *E. coli* bacteriuria is often treated with antibiotics independent of any symptoms [19]. Overtreatment in turn generates added cost and can facilitate the development of antibiotic resistance. It would therefore be invaluable if we had tools to differentiate between symptomatic infection and asymptomatic bacteriuria (and to understand which bacteriurias result in morbidity in patients who are unable to report symptoms). Here, we report a comparison of patient characteristics and virulence factors between symptomatic and asymptomatic patients with *E. coli* bacteriuria. While we found independent clinical predictors for asymptomatic bacteriuria (chronic pulmonary disease, dementia) no virulence pattern could be associated with the corresponding *E. coli* isolates.

To our knowledge there are no published studies of patient characteristics to differentiate ASB from symptomatic UTI. We found the comorbidities chronic pulmonary disease and dementia to be associated with asymptomatic bacteriuria. It is conceivable that underlying dementia resulted either in poor symptom recognition by the affected patients or may have impaired their ability to report symptoms. ASB is also much more prevalent in elderly long-term care residents [20] who are prone to cognitive deficits, and cognitive changes may be a risk factor for ASB [21]. Other potential contributors are poor perineal hygiene and incomplete bladder emptying. It is less clear why chronic pulmonary disease should be associated with ASB; this may be due to different screening practices in medical subspecialties or a spurious finding. Those with ASB may finally represent a group of patients in which it was more difficult to find a diagnosis and urine cultures were part of the broad work-up.

A small number of studies have determined differences in bacterial virulence factors between strains causing ASB and sUTI. Mabbett et al. compared 57 ASB strains to 45 cystitis/pyelonephritis strains not only in regard to their virulence genes but also in gene product expression and epithelial adhesion studies [8]. *PapGII* (47% vs. 16%) and *afa* (29% vs. 11%) were associated with sUTI as were hemolysin expression, siderophore expression, and adhesion to epithelial cells. However, the authors noted that ASB and sUTI strains tended to be phylogenetically related. The latter finding was confirmed by Takahashi et al. who investigated virulence factors in 283 *E. coli* urinary isolates from a hospital in Japan, and also saw a similar distribution of serotypes across ASB and sUTI isolates. The virulence genes *iha* (31% vs. 13%), *ompT* (82% vs. 70%) and *PAI*, which refers to the so-called genomic “pathogenicity island” (65% vs. 50%), were all significantly more frequent in strains causing sUTI [9]. Although *ompT* was a common virulence gene in our similar-sized cohort, the sUTI strains we analyzed did not carry *ompT* more frequently (83% vs. 76%). A number of smaller studies in subpopulations like pregnant women and patients with spinal cord injuries found other virulence factors to be associated with symptomatic infections [10,11] or were negative [22]. In addition to the bacterial characteristics mentioned above, there have been reports that ASB strains can be identified based upon their capability to form biofilms *in vitro*[23], for which we did not screen our isolates. With 287 patient samples our cohort is one of the largest to date; however, we did not find any virulence gene to predominate in the subset of symptomatic patients. One of the possible explanations for this negative finding is that virulence genes in ASB may carry deletions that block gene expression [24-26]; therefore, ASB strains may look more virulent by genotype than they actually are *in vivo*. Another possibility is that virulence factors other than those we examined are more relevant in determining pathogenicity [27]. Yet another possibility is that the major determinant of symptom development is unrelated to pathogen factors, but due to unmeasured factors such as the host’s innate immune response [28-30]. Future research should incorporate studies of the host’s defense mechanisms (e.g., toll-like receptor polymorphisms) into a multi-faceted analysis of symptom development in bacteriuria.

There are a number of limitations to our study. Including only patients that were tested for and found to have bacteriuria may have introduced selection bias. We suspect that this bias was particularly relevant for ASB patients which were included despite an indication for culturing. Symptoms at the time of bacteriuria were taken from hospital charts entered by physicians and nurses; therefore patient symptoms may have been underreported. We did not obtain urine samples to determine whether virulence factors were expressed *in vivo,* nor test gene expression of the bacterial isolates *in vitro.* Also, we elected to test for a specific set of virulence factors but may have missed others that are not identified yet. Blood cultures were ordered by the treating physicians and may have missed septic or febrile patients at risk for bacteremia. Lastly, we did this study at a single tertiary-care center and the findings may not be generalizable to other settings. This also goes for the high levels of resistance to the most commonly used antimicrobials that we encountered here.

## Conclusions

In summary, our study is the largest cohort study to analyze both patient and pathogen factors associated with asymptomatic versus symptomatic bacteriuria, was conducted prospectively and included both genders. Novel predictive factors (chronic pulmonary disease, dementia) were identified that could serve for clinical decision-making on antibiotic treatment. Host susceptibility (i.e., characteristics of an individual’s innate immune system) may be more relevant for symptom development in *E. coli* bacteriuria than virulence determinants.

## Competing interest

None of the following authors has a conflict of interest (J Marschall: no conflict, ML Piccirillo: no conflict, JP Henderson: no conflict). DK Warren is a Consultant for 3 M Healthcare and Cardinal Health, Inc., and receives research funding from Sage Products, Inc., 3 M Healthcare, BioMérieux, and Cubist Pharmaceuticals.

## Authors’ contributions

JM conceived and designed the study, did the statistical analyses, and drafted the manuscript. MLP abstracted the clinical data and helped draft the manuscript. BF and LZ oversaw the laboratory analyses and reviewed the manuscript. DKW and JPH helped design the study and reviewed the manuscript. All authors read and approved the final version of the manuscript.

## Pre-publication history

The pre-publication history for this paper can be accessed here:

http://www.biomedcentral.com/1471-2334/13/213/prepub
